# *Fusarium kuroshium* is the primary fungal symbiont of an ambrosia beetle, *Euwallacea fornicatus*, and can kill mango tree in Japan

**DOI:** 10.1038/s41598-023-48809-8

**Published:** 2023-12-07

**Authors:** Zi-Ru Jiang, Momo Tanoue, Hayato Masuya, Sarah M. Smith, Anthony I. Cognato, Norikazu Kameyama, Keiko Kuroda, Hisashi Kajimura

**Affiliations:** 1 Laboratory of Forest Protection, Graduate School of Bioagricultural Sciences, Nagoya University, Nagoya, 464-8601 Japan; 2 School of Agricultural Sciences, Nagoya University, Nagoya, 464-8601 Japan; 3 Department of Forest Microbiology, Forestry and Forest Products Research Institute, Tsukuba, 319-1301 Japan; 4 Department of Entomology, Michigan State University, East Lansing, MI 48824 USA; 5 Faculty of Agriculture, University of the Ryukyus, Okinawa, 903-0213 Japan; 6 Graduate School of Agricultural Science, Kobe University, Kobe, 657-0013 Japan

**Keywords:** Microbial communities, Fungi, Pathogens

## Abstract

This study identifies fungi associated with *Euwallacea fornicatus* and determines whether these fungal species play the role of primary symbiont. *E. fornicatus* adults that emerged from the branches of infested trees in Okinawa main island, Japan, were collected and used to isolate fungi. *Fusarium kuroshium* and *Penicillium citrinum* were the most dominant fungal associates of females and males, respectively. *F. kuroshium* was much more frequently isolated from the head, including mycangia (fungus-carrying organs), of females than any other body parts. We inoculated healthy mango saplings with *F. kuroshium* or *F. decemcellulare*, both of which were symbionts of *E. fornicatus* females infesting mango trees. *F. kuroshium* decreased leaf stomatal conductance and rate of xylem sap-conduction area and increased length and area of xylem discoloration of the saplings, thereby weakening and killing some. These results suggest that *F. kuroshium*, a mycangial fungus of *E. fornicatus*, inhibits water flow in mango trees. This study is the first to report that *F. kuroshium* causes wilt disease in mango trees and that it is a primary fungal symbiont of *E. fornicatus*.

## Introduction

The ambrosia beetle, a group of wood-boring mycetophagous insects belonging to subfamilies Scolytinae and Platypodinae in the Curculionidae family, provides a prime example of fungus-insect symbiosis^1,2^. The adult female of these species possesses mycangia (fungus-carrying structures)^3^ which carry fungal spores that can be stored in natal galleries of host trees as food for larvae^4,5^. However, some fungal symbionts are phytopathogenic and cause harm to the trees^6–9^. These pathogens tend to occupy new habitats established by their vector beetles and are often lethal to host trees^10,11^. International trade is one of the main factors responsible for globally spreading many ambrosia beetles^12–14^.

The shot hole borer (SHB) of *Euwallacea* ambrosia beetles (Scolytinae)-*Fusarium* dieback (FDB) is a pest-disease combination affecting various tree species in many countries and regions^8,15,16^. SHB taxa include species such as (i) tea shot hole borer (TSHB), (ii) polyphagous shot hole borer (PSHB), and (iii) kuroshio shot hole borer (KSHB), their corresponding scientific name are: *E. perbrevis* Schedl, *E. fornicatior* Eggers, *E. fornicatus* Eichhoff, and *E. kuroshio* Gomez et Hulcr, respectively^16^ (Table 1). These four *Euwallacea* species, which are termed the *E. fornicatus* species complex, when unidentifiable, exhibit a broad host range involving a total of 412 plant species in 75 families^17^, including important crops, such as tea (*Camellia sinensis* L.)^18^, avocado (*Persea americana* Mill.)^8,15,19,20^, box-elder (*Acer negundo* L.), castor bean (*Ricinus communis* L.), English oak (*Quercus robur* L.)^15,20^, and London plane (*Platanus acerifolia* Willd.)^21^ (Table 1). All four *Euwallacea* species possess oral mycangia^3^ and act as vectors of members of the Ambrosia *Fusarium* Clade^22,23^.

**Table 1:** Worldwide summary of *Euwallacea fornicatus* species complex–*Fusarium* sp. symbiosis in relation to tree damage.

Beetle species	Fungal species	Host trees	Country (region)	References
*Euwallacea perbrevis* (Schedl) = Tea shot hole borer (TSHBa)	*Fusarium rekanum* Lynn et Marinc	*Acacia crassicarpa* A. Cunn. ex Benth.	Indonesia (Riau)	^53^
*Euwallacea fornicatior* (Eggers) = Tea shot hole borer (TSHBb)	*Fusarium ambrosium* Gadd et Loos	Chinese tea (*Camellia sinensis* L.); Avocado (*Persea americana* Mill.)	India, Sri Lanka, USA (Florida, California), Israel	^7,8,18,19^
*Euwallacea fornicatus* (Eichhoff) = Polyphagous shot hole borer (PSHB)	*Fusarium euwallaceae* Freeman et al.	Numerous woody hosts	Israel, USA (California, Los Angeles), South Africa	^15,21,35,54^
*E. fornicatus* (Eichhoff) = Polyphagous shot hole borer (PSHB)	*Fusarium kuroshium* Na et al.	Mango (*Mangifera indica* L.)	Japan (Okinawa)	This study
*Euwallacea kuroshio* Gomez et Hulcr = Kuroshio shot hole borer (KSHB)	*F. kuroshium* Na et al.	California sycamore (*Platanus racemose* Nutt.); Avocado (*Persea americana* Mill.)	USA (California), Mexico, Taiwan	^23,24,36,55^

*Fusarium kuroshium* Na et al., obtained from *E. kuroshio*^24^ is related to, but distinct from *F. euwallaceae* Freeman et al., which is associated with *E. fornicatus*^15^ (Table 1). *F. kuroshium* acts as a causal agent of FDB in several tree species due to its accelerated growth in wood tissue, which blocks xylem vessels and obstructs water flow in trees^24–26^. Internal symptoms characteristic of FDB manifest as reddish-to-dark brown lesions (and its variations) in the xylem^8^ and reduced stomatal conductance and net photosynthetic rates of leaves and plant biomass^27^.

In Japan, *E. fornicatus* (the name formerly used before being categorized as a species complex) was first recorded on *Leucaena glauca* (Benth) on Chichi-jima Island in 1973^28^. In 2000, *E. fornicatus* was declared as a pest of mango (*Mangifera indica* L.) trees in Tokuno-shima Island^29^. Since 2007, this pest has been causing severe damage to mango orchards in Okinawa, the main island^30^. However, despite the threats posed to the mango industry, the causal relationship between *E. fornicatus* infestations and the decline in mango trees remain unclear. This is mainly attributed to the lack of clarity regarding the role played by the fungus as a link between the borer and mango trees.

Therefore, in this study, we identify the pest species and clarify the associated fungal flora to determine the primary symbiont, with particular reference to mycangial fungi. Moreover, to demonstrate a causal relationship, we assessed the pathogenicity of the symbiont in relation to mango trees.

## Results

### Beetle collection and identification

A total of 130 beetle specimens (♀ = 85; ♂ = 45) were collected from July 31 to October 12, 2018. A literature survey^23^ confirmed that the morphological characteristics of all examined beetles (Supplementary Fig. S1b; ♀) were consistent with those of *E. fornicatus* (PSHB).

**Figure 1:**
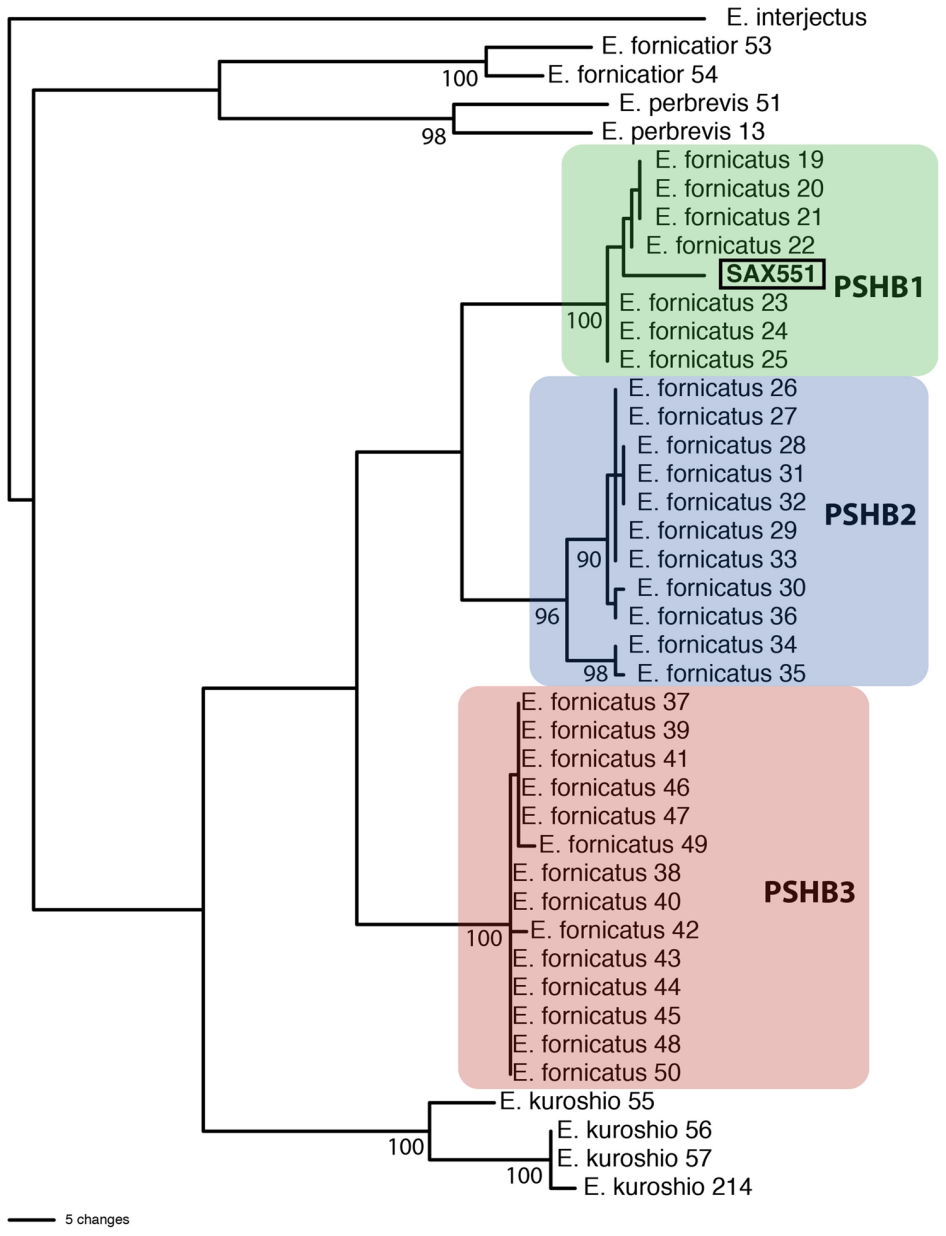
Phylogenetic placement of the sampled female beetle (SAX551) based on COI and CAD DNA sequences confirming its identity as *Euwallacea fornicatus*. One of 36 most parsimonious trees demonstrates the relationship of SAX551 among other *Euwallacea* species and its placement in the PSHB1 clade. Numbers after species names correspond to the last two digits of Genbank numbers. Numbers at nodes are bootstrap values.

The phylogenetic analysis returned 36 most parsimonious trees which differed only in the placement of some conspecific individuals (Fig. 1). Species and the three major lineages of PSHB were monophyletic with bootstrap values > 96. The individual sampled from this study (SAX551) was confirmed as *E. fornicatus* and as a member of the PSHB1 clade (as in Wang et al.^31^) which supports the morphological identification.

### Fungal flora

A total of 512 isolates were purified: 97, 118, and 122 isolates were obtained from the head, thorax, and abdomen of females, respectively, while 56, 60, and 59 isolates were from head, thorax, and abdomen of males, respectively (Table 2). Following morphological categorization, 66 selected isolates were sequenced. Finally, 23 fungal isolates with varying compositions were identified as 20 species from females and 15 species from males, including two identical species in both sexes. The sequences of eight isolates failed to amplify via PCR, and these were treated as unknown species (Table 2).

**Table 2:** Fungal species relative dominance (RD%) and frequency of occurrence (FO%) on adults of *Euwallacea fornicatus*.

Fungal species^c^	No. of fungal isolates and relative dominance (RD, %) ^a^ in each body part					
	**♀**			**♂**		
	Head	Thorax	Abdomen	Head	Thorax	Abdomen
*Fusarium kuroshium*	76 (78.4)	17 (14.4)	20 (16.4)	8 (14.3)		2 (3.4)
*Penicillium citrinum*		32 (27.1)	37 (30.3)	21 (37.5)	20 (33.3)	19 (32.2)
*Candida insectorum*		8 (6.8)	3 (2.5)	1 (1.8)		1 (1.7)
*Phialemoniopsis curvata*			1 (0.8)	5 (8.9)	15 (25.0)	3 (5.1)
*Aspergillus tubingensis*		4 (3.4)	2 (1.6)			
*Fusarium decemcellulare*		5 (4.2)	1 (0.8)			
*Lasiodiplodia theobromae*	3 (3.1)	7 (5.9)	11 (9.0)			
*Paracremonium* sp.		10 (8.5)	7 (5.7)			
*Chaetomium cruentum*				3 (5.4)	3 (5.0)	
*Chaetomium globosum*				2 (3.6)		
*Talaromyces purpureogenus*				4 (7.1)	4 (6.7)	1 (1.7)
*Paecilomyces formosus*		2 (1.7)	1 (0.8)			
*Aspergillus tamarii*		2 (1.7)				
*Candida jaroonii*		1 (0.8)				
*Fusarium proliferatum*			1 (0.8)			
*Graphium jumulu*	1 (1.0)					
*Gibberella intricans*			1 (0.8)			
*Penicillium copticola*		1 (0.8)				
*Purpureocillium lilacinum*			1 (0.8)			
*Aspergillus* sp.					1 (1.7)	
*Phaeoacremonium scolyti*					1 (1.7)	
*Pseudopithomyces chartarum*					1 (1.7)	
*Pseudocosmospora vilior*				1 (1.8)	1 (1.7)	
Unknown-1	4 (4.1)	1 (0.8)				
Unknown-2	4 (4.1)	5 (4.2)	3 (2.5)			
Unknown-3	6 (6.2)	19 (16.1)	21 (17.2)			
Unknown-4	3 (3.1)	4 (3.4)	12 (9.8)			
Unknown-5				4 (7.1)	6 (10.0)	6 (10.2)
Unknown-6				4 (7.1)	4 (6.7)	8 (13.6)
Unknown-7				3 (5.4)	3 (5.0)	19 (32.2)
Unknown-8					1 (1.7)	
Total number of fungal isolates of all the species	97	118	122	56	60	59

Fungal species^c^	No. of beetles from which each fungal species was isolated and frequency of occurrence (FO, %)^b^ in each body part					
	♀			♂		
	Head	Thorax	Abdomen	Head	Thorax	Abdomen
*Fusarium kuroshium*	76 (89.4)	17 (20.0)	20 (23.5)	8 (17.8)		2 (4.4)
*Penicillium citrinum*		32 (37.6)	37 (43.5)	21 (46.7)	20 (44.4)	19 (42.2)
*Candida insectorum*		8 (9.4)	3 (3.5)	1 (2.2)		1 (2.2)
*Phialemoniopsis curvata*			1 (1.2)	5 (11.1)	15 (33.3)	3 (6.7)
*Aspergillus tubingensis*		4 (4.7)	2 (2.4)			
*Fusarium decemcellulare*		5 (5.9)	1 (1.2)			
*Lasiodiplodia theobromae*	3 (3.5)	7 (8.2)	11 (12.9)			
*Paracremonium* sp.		10 (11.8)	7 (8.2)			
*Chaetomium cruentum*				3 (6.7)	3 (6.7)	
*Chaetomium globosum*						
*Talaromyces purpureogenus*				4 (8.9)	4 (8.9)	1 (2.2)
*Paecilomyces formosus*		2 (2.4)	1 (1.2)			
*Aspergillus tamarii*		2 (2.4)				
*Candida jaroonii*		1 (1.2)				
*Fusarium proliferatum*			1 (1.2)			
*Graphium jumulu*	1 (1.2)					
*Gibberella intricans*			1 (1.2)			
*Penicillium copticola*		1 (1.2)				
*Purpureocillium lilacinum*			1 (1.2)			
*Aspergillus* sp.					1 (2.2)	
*Phaeoacremonium scolyti*					1 (2.2)	
*Pseudopithomyces chartarum*					1 (2.2)	
*Pseudocosmospora vilior*				1 (2.2)	1 (2.2)	
Unknown-1	4 (4.7)	1 (1.2)				
Unknown-2	4 (4.7)	5 (5.9)	3 (3.5)			
Unknown-3	6 (7.1)	19 (22.4)	21 (24.7)			
Unknown-4	3 (3.5)	4 (4.7)	12 (14.1)			
Unknown-5				4 (8.9)	6 (13.3)	6 (13.3)
Unknown-6				4 (8.9)	4 (8.9)	8 (17.8)
Unknown-7				3 (6.7)	3 (6.7)	19 (42.2)
Unknown-8					1 (2.2)	
Total number of beetles tested	85	85	85	45	45	45

^a^RD (%) = Number of fungal isolates of each species/Total number of fungal isolates of all the species × 100%

^b^FO (%) = Number of beetles from which each fungal species was isolated/Total number of the beetles used for isolation × 100%

^c^The sequences of eight isolates failed to amplify via PCR, and these were treated as unknown species.

### Phylogenetic analysis for *Fusarium* fungi

**Figure 2:**
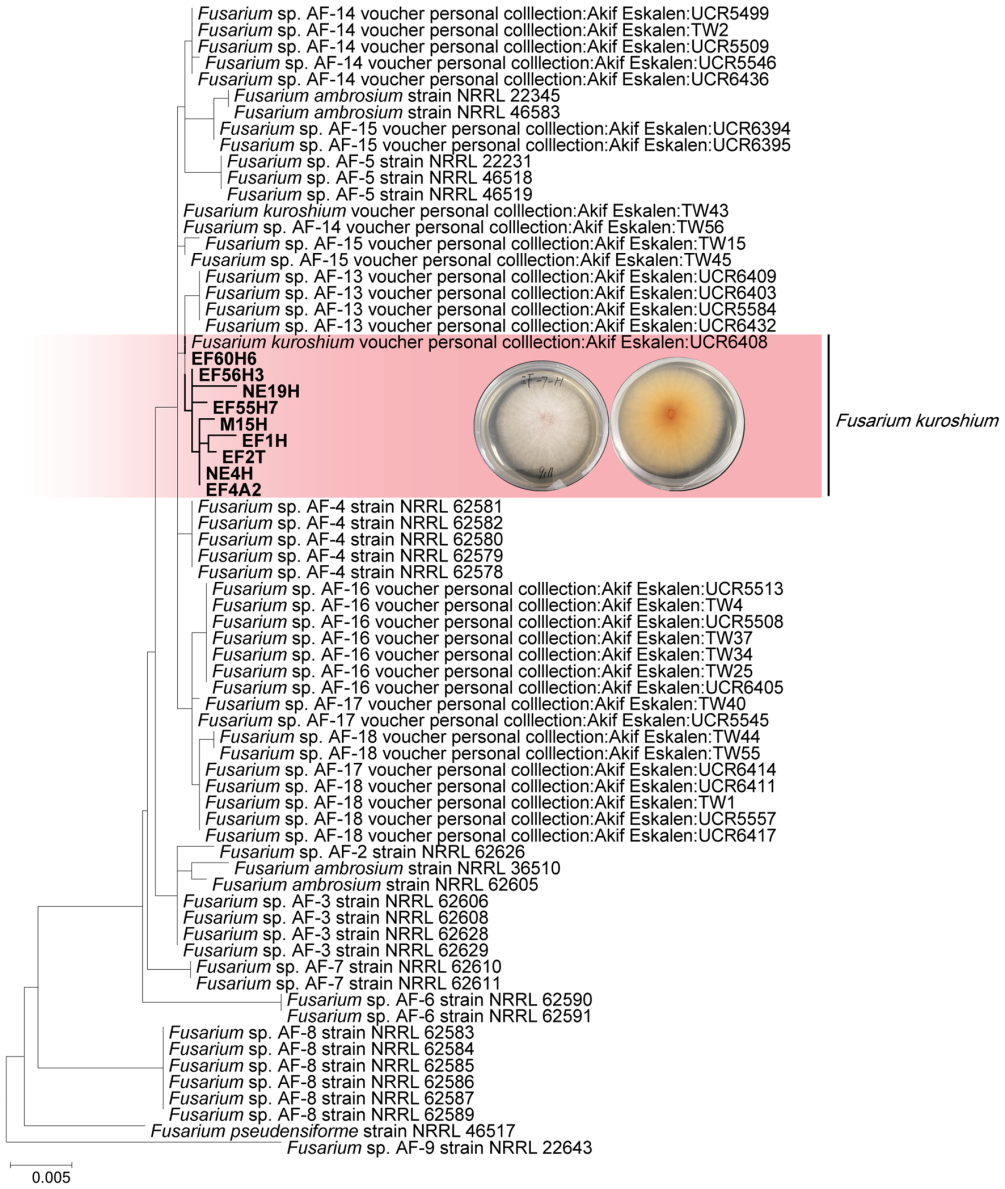
Phylogenetic placement of *Fusarium* species isolated from the head and oral mycangia of female adults of *Euwallacea fornicatus*. Fungal isolates obtained in this study are in a bold and red background. The phylogeny tree was constructed using the maximum likelihood method based on the Kimura 2-parameter model with MEGA7. The tree involved 70 nucleotide sequences. All positions with less than 95% site coverage were eliminated; fewer than 5% alignment gaps, missing data, and ambiguous bases were allowed at any position. A total of 1745 positions were present in the final dataset.

The DNA sequencing data consisted of a total of 1,745 positions. *Fusarium pseudensiforme* (NRRL 46517) and *Fusarium* sp. [AF-9] (NRRL22643) were used as the outgroup^32,33^. According to the phylogenetic tree, the *Fusarium* species isolated from the head, including oral mycangia of *E. fornicatus*, was placed together with *F*. *kuroshium* in an independent clade within the ambrosia fusaria (Fig. 2). Therefore, we identified the ambrosia fusaria associated with *E. fornicatus* infesting mango tree in this study as *F*. *kuroshium*.

### Relative dominance and frequency of occurrence

**Figure 3:**
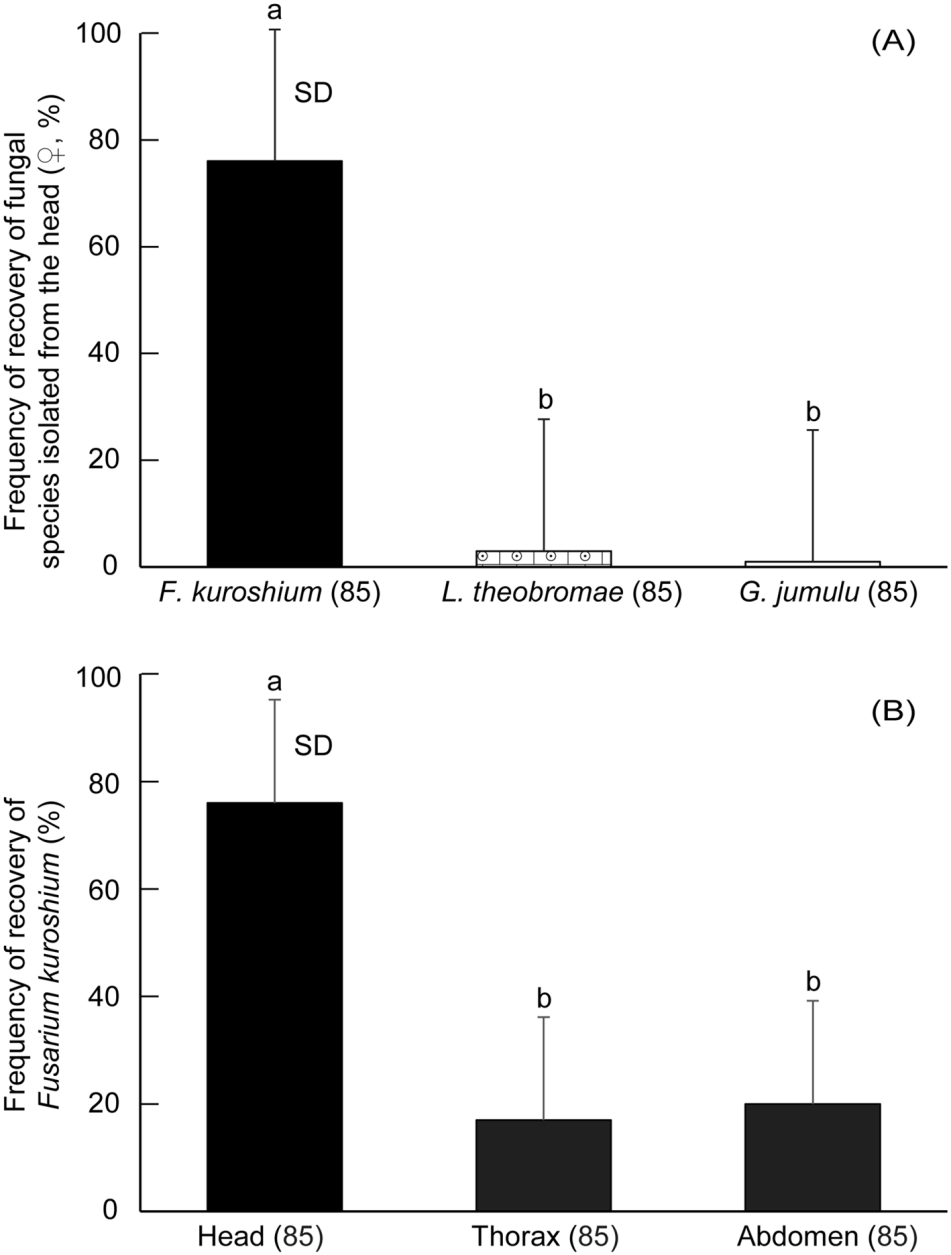
Frequency of occurrence of fungal species isolated from the head, thorax, and abdomen of female adults of *Euwallacea fornicatus*. Figures at A and B compare the frequencies of fungal species and the frequencies of *Fusarium kuroshium* among various body parts, respectively. The figure in parentheses indicates the number of beetles tested. Mean frequencies with different letters are significantly different among fungal species or body parts at the 1% level, using Fisher’s exact test with Bonferroni correction. SD, standard deviation.

In females, most isolates belonging to *F. kuroshium* showed the highest relative dominance (RD) (78%) in the head, with RD in the thorax and abdomen being 14.4% and 16.4%, respectively (Table 2). The frequency of occurrence (FO) of *F. kuroshium* in the head (89.4%) was much higher than that of other fungi in the head (1.2–43.5%), while its FO in the thorax and the abdomen were 20.0% and 23.5%, respectively (Table 2); these differences were statistically significant (Fig. 3). Another *Fusarium* fungus, *F. decemcellulare*, was found in thorax and abdomen of females, but with a lower RD and a lower FO (Table 2).

*Penicillium citrinum* was a commonly detected species, with FOs of 37.6% and 43.5% in the thorax and abdomen of females, respectively (Table 2). However, the FO of *P. citrinum* in the head of females was never detected, although in males, it was dominant in all body parts compared with the females (Table 2).

### External symptom

**Figure 4:**
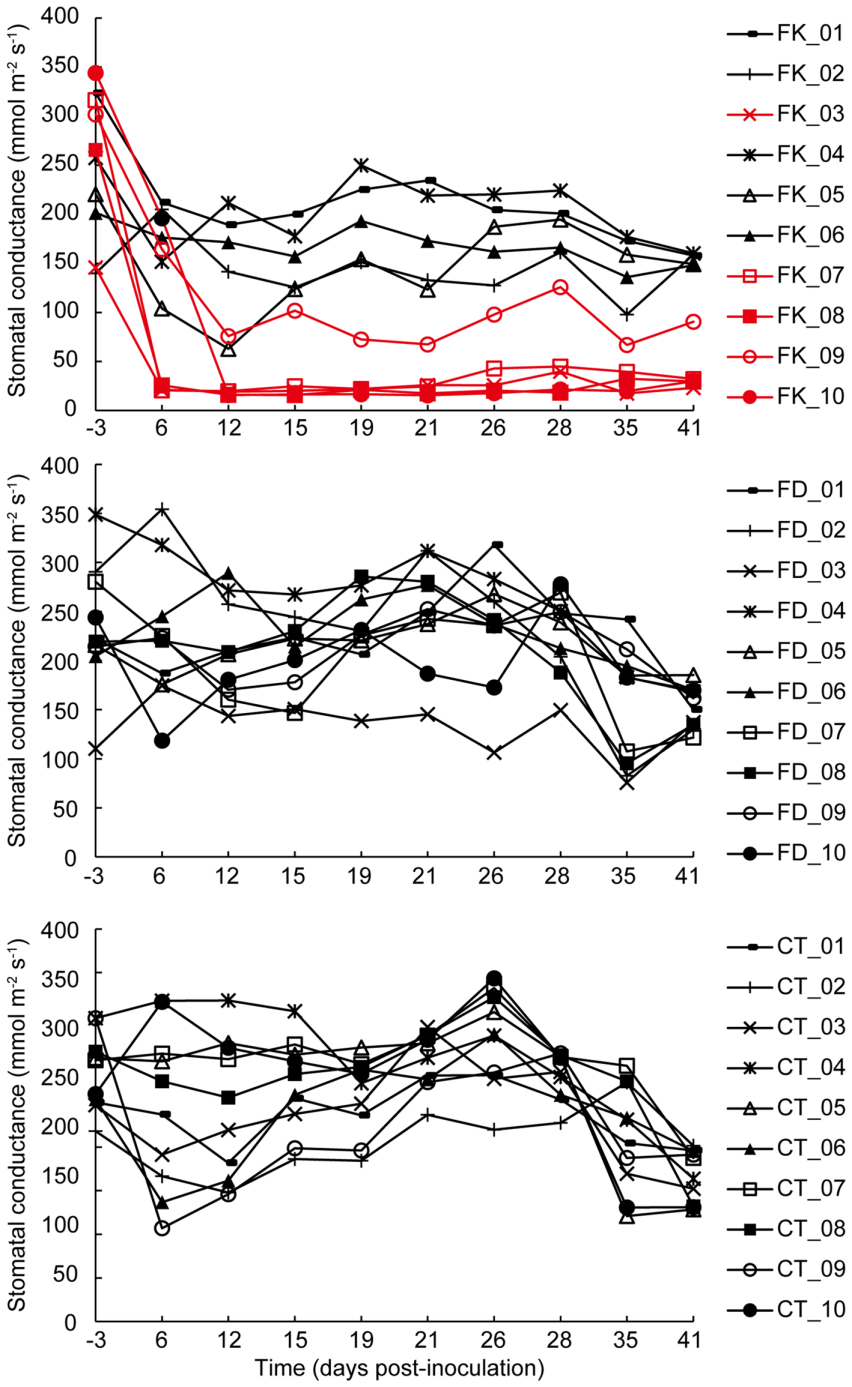
Mean leaf stomatal conductance in each *Mangifera indica* sapling. Means were calculated from data obtained via measuring the same 5 leaves twice a week between 9:00 and 13:00 h using a leaf porometer. FK *F. kuroshium*, with four saplings (red line) that eventually perished. FD *F. decemcellulare*; CT sterilized control toothpick. The treatment information is shown in Table 3.

The initial change observed was that several saplings subjected to the toothpicks inoculated with *F. kuroshium* (FK) treatment started to wilt rapidly around 3 days following inoculation, and 4 saplings (FK_3, 7, 8, 10; referred to as FK_D) finally died (Supplementary Figs. S1 and S4). The other 6 saplings (FK_1, 2, 4, 5, 6, 9; referred to as FK_L) survived until the end of monitoring (Supplementary Fig. S4). No wilting was observed on all saplings subjected to the toothpicks inoculated with *F. decemcellulare* (FD) and sterilized control toothpicks (CT) treatments (Supplementary Fig. S4). After inoculation, leaf stomatal conductance (LSC) in FK_D and FK_5 and 9 decreased markedly, with FK_5 and 9 showing slight recovery, whereas LSCs in the other FK, FD, and CT treatments maintained over 100 of its value throughout monitoring (Fig. 4).

### Internal symptoms

**Figure 5:**
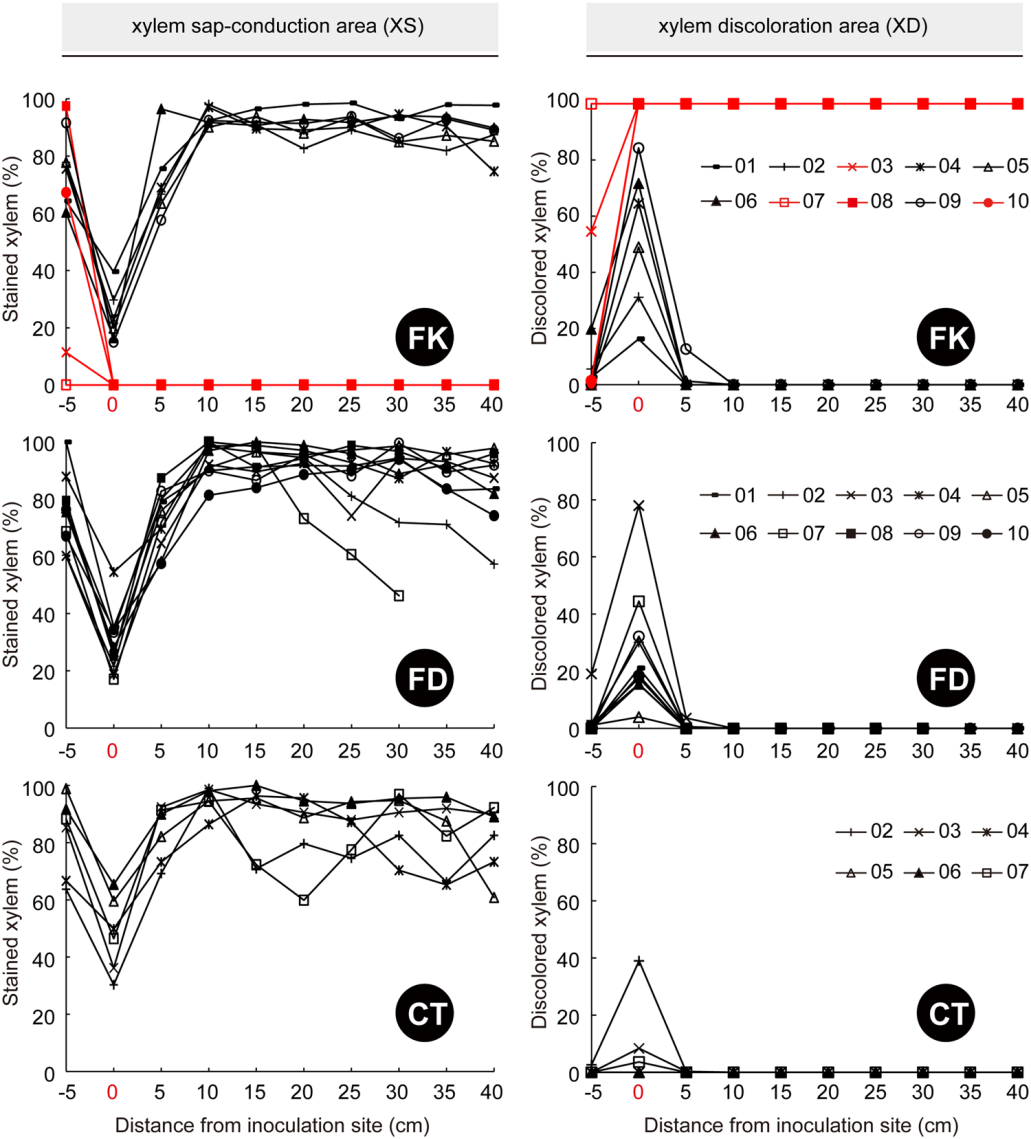
Rates of xylem sap-conduction and xylem discoloration in each *Mangifera indica* sapling. FK *F. kuroshium*, with four saplings (red line) that eventually perished. FD *F. decemcellulare*; CT sterilized control toothpick. The treatment information is shown in Table 3.

In contrast to FD and CT, no pink area above the inoculation site was observed in FK_D (Supplementary Fig. S5). The rates (percent ratios) of xylem sap-conduction area (XS) values (Fig. 5) of FK_D were 0% at 0–40 cm distance from the site, whereas those of FK_L, FD, and CT mostly ranged between 60% and 100%, except for the site representing 0 cm. In addition, a pink area was observed at a − 5 cm distance from the site, even in FK_D (Supplementary Fig. S5), while the two saplings of FK_D had over 60% XS, as did FD and CT (Fig. 5). These findings indicated that water had flowed from roots to the upper stems, passing through the narrow but still functional zones of xylem.

Brown areas were clearly observed in FK_D overall, as also in FD at the site (0 cm) (Supplementary Fig. S5). As evidence of this, rates (percent ratios) of xylem discoloration area (XD) in FK_D were 100%, except at − 5 cm distance from the site in three saplings; furthermore, at 0 cm, those in most of FK_L and two of the FDs were over 40% (Fig. 5).

**Figure 6:**
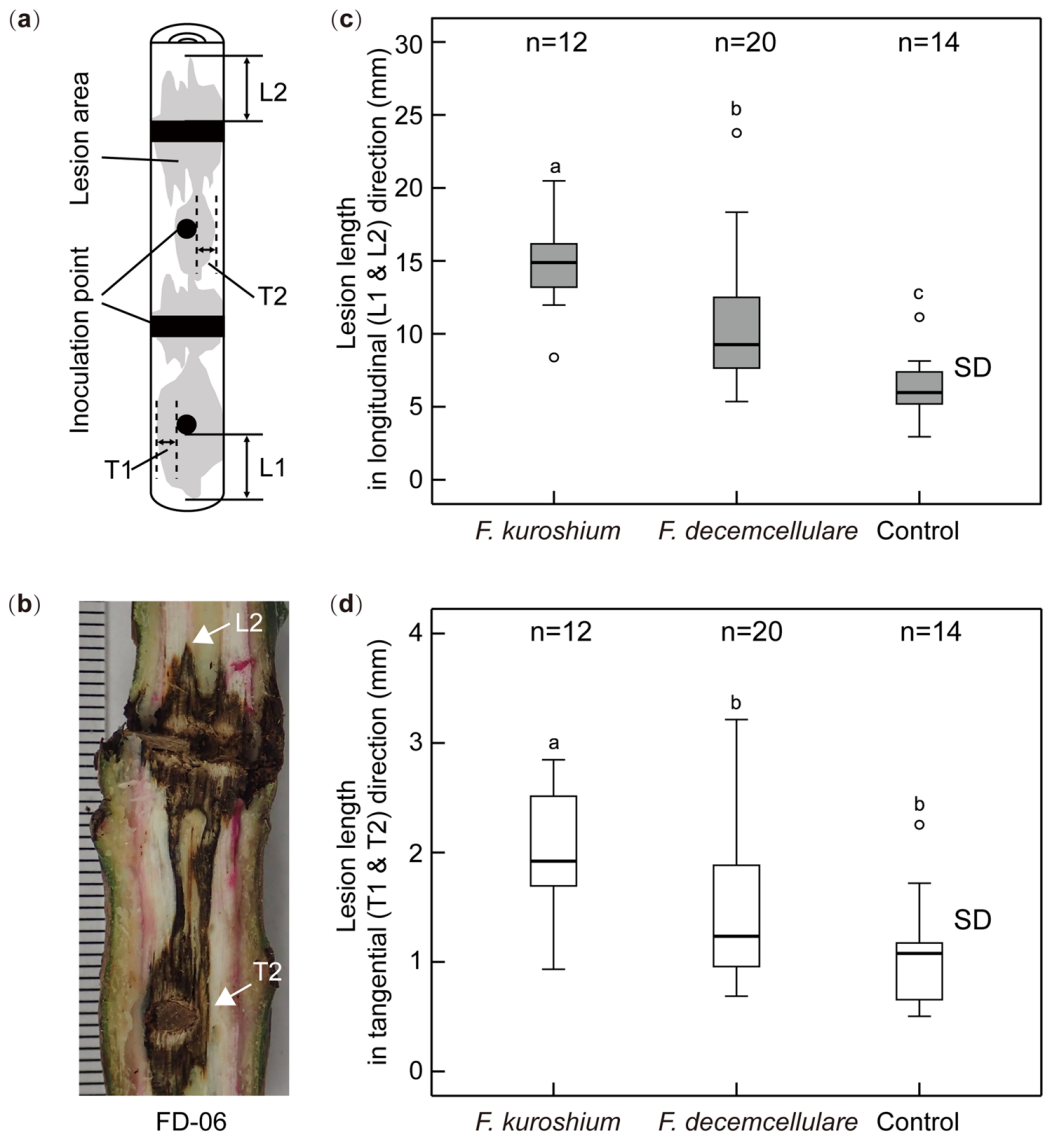
Lesion lengths showing xylem discoloration caused by *Fusarium kuroshium*, *Fusarium decemcellulare*, and a sterilized toothpick (control). (**a**,**b**) show measurement directions (L: longitudinal; T: tangential) of lesion length. If lesions between inoculation sites were connected, the data were not used. (**c**,**d**) show box (25–75% data range) and whisker (values within 1.5 interquartile range) plots of the length in longitudinal and tangential directions, respectively. Mean lengths with different letters are significantly different among treatments at the 1% level; Kruskal–Wallis test with Bonferroni correction; *SD* standard deviation.

Lesions caused by the fungal inoculum and drilling wounds spread from the inoculation site (Fig. 6). In FK_D, the lesion area could no longer be recognized due to it being masked by necrosis. Therefore, the lesion measurements of FK_L were compared with those of FD and CT. Average lesion lengths in the longitudinal direction, were significantly longer in FK_L (1.47 cm), FD (1.04 cm), and CT (0.63 cm), in that order (Fig. 6c). Moreover, in the tangential direction, the average of FK_L was significantly longer than that of FD and CT, both of which showed no significant difference (Fig. 6d).

In re-isolation, *F. kuroshium* was certain to be found in stem tissues around inoculation sites of FK_D. By contrast, fungal inocula were not detected in any other saplings.

## Discussion

In this study, we define a ‘symbiotic’ relationship as a close and long-term biological interaction between ambrosia beetles and their fungal associates. These fungal associates must be stored in the beetles’ mycangia before being released into the galleries, and they should represent a significant proportion of the fungal flora collected from the mycangia. Fungi associated with ambrosia beetles were classified into primary ambrosia fungi (PAF) and auxiliary ambrosia fungi (AAF)^34^. The findings of this study indicated that, of the 23 species of fungi that were identified, *F. kuroshium* was most closely associated with *E. fornicatus* females and dominant in its head. Thus, the female, which has oral mycangia in its head^3^, serves as the vector for *F. kuroshium* (Table 2), which is a PAF. The nutritional role played by *F. kuroshium*, the spores and/or hyphae of which serves as a food source for *E. fornicatus* larvae, remains to be analyzed. The other fungal species found in the head of *E. fornicatus* showed far lower dominance and were isolated from only a few beetle samples, thus being regarded as AAF. By contrast, in *E. fornicatus* males, *P. citrinum* was more frequently isolated than other species (Table 2), suggesting that a lack of mycangia may strongly affect the abundance and dominance of the fungal flora.

*Fusarium* fungus-*Euwallacea* beetle symbiosis has been reported in many countries and regions (Table 1) but with high levels of variation between them^23,35,36^. *F. kuroshium* has been isolated from the heads of *E. kuroshio*, which attacks California sycamore (*Platanus racemosa* Nutt.) and avocado (*P. americana*) in California, United States^23,24^ (Table 1). However, our results indicated that *F. kuroshium* is associated with *E. fornicatus* in Okinawa main island, Japan. Previous studies have shown that, although a strict relationship exists between them in native areas^36^, the relationship becomes unstable in invasion areas, due to “host shifts”, which are attributed to *Fusarium* fungi being able to change host beetles over time^16,35^. Several exotic species have switched or gained fungal associates in areas they were newly introduced^36,37^. Co-phylogenetic analyses have been conducted to assess symbiont fidelity within these symbioses^35^. Thus, although *F. kuroshium*-*E. fornicatus* symbiosis in Okinawa may remain similar in origin, it may have possibly been switched during adaptation to a new habitat, namely mango orchards. Furthermore, recent studies have revealed that *E. fornicatus* and *E. kuroshio* can reproduce and survive on each other’s symbiotic fungi in their invasive range on artificial media^36^, although normally *E. fornicatus* does not reproduce well in avocado^38^. Our study indicates that these possibilities remain unresolved.

*F. kuroshium* is the causal agent of FDB in several tree species, in which the fungus grows into wood tissue, blocks xylem vessels, and obstructs water flow throughout the host plant^8,24,39^. To the best of our knowledge, this study is the first to demonstrate the pathogenicity of *F. kuroshium* in mango. Herein, 40% of the *F. kuroshium*-inoculated saplings died with symptoms similar to those seen in mango orchards, which indicated that xylem sap-conduction, as well as leaf stomatal conductance and xylem coloration, were seriously compromised. Our inoculation tests clarified that *F. kuroshium* caused the largest tangential and longitudinal lesions, which tend to destroy xylem parenchyma cells via mycelial invasion^40^. Thus, the novel FDB observed in mango in Okinawa, Japan following *E. fornicatus* migration may be attributed to a unique beetle-fungus-tree combination. This is similar to *Fusarium* spp.-*E. fornicatus* symbiosis in wilt syndrome and avocado trees seen in Israel^41^.

Conversely, *F. decemcellulare* appears to be a ‘by-chance’ species in the thorax and abdomen of *E. fornicatus*, being classified as AAF (Table 2). A pathogenicity study conducted in Puerto Rico^42^ found that saplings inoculated with *F. decemcellulare* showed no FDB and displayed lesions that were significantly larger than those of the control but smaller than those in saplings inoculated with *F. kuroshium*. These results suggested that *F. decemcellulare* may have weak pathogenic potential pertaining to mango. However, according to a study conducted by Qi et al.^43^ in China, *F. decemcellulare* did cause dieback in *M. indica* ‘Keitt’, which is a variety that is different from the one (*M. indica* ‘Irwin’) used in this study. Therefore, more detailed studies to evaluate the susceptibility of different varieties to fungal pathogenicity and beetle-linked boring may be warranted.

Our study also revealed that *Lasiodiplodia theobromae* (Pat.) Griffon & Maubl., classified as AAF, consistently appeared in the females of all three body parts (Table 2). Previous studies have reported that *L. theobromae* caused serious disease in mango trees in Sindh, Pakistan^44^. This discovery suggests that this fungal pathogen may potentially have risk for wilt disease in mango trees also in Japan. To address this question, further inoculation experiments involving this fungus on mango trees or saplings are required.

## Conclusions

This study found that *F. kuroshium*, a mycangial fungus of *E. fornicatus*, may be a pathogen of mango, whereas *F. decemcellulare* only showed weak pathogenic potential with respect to mango. Our findings provide strong evidence that *F. kuroshium*-*E. fornicatus* symbiosis causes mango wilt disease at least in Okinawa main island, Japan, and may thereby contribute to target detection for wilt disease management.

## Materials and methods

### Sample collection

In the summer of 2018, *M. indica* (variety; Irwin) trees showing wilting and discoloration of leaves were found in a mango orchard in Nago city, Okinawa main island, Japan. The trees displayed numerous small holes on the surfaces of their trunks and branches, and these holes were found extruding wood particles (Supplementary Fig. S1a). This infestation pattern was typical of boring by ambrosia beetles.

A representative 50-cm long log (Supplementary Fig. S1a) was cut from an infested branch on August 30, 2018 and rapidly transported to the laboratory of Nagoya University. The log was placed in a container, which was then sealed, to capture emerging adult beetles. The adults were collected every few days and immediately placed in 1.5-mL sterile tubes using sterilized forceps for fungal isolation at a later stage.

All collected adults were observed under stereo microscopes (OLYMPUS SZ6045-TRPT and SZX16) (Olympus Optical Co., Ltd., Tokyo, Japan) and identified using morphological features^23^. Some of them were forwarded to Michigan State University for confirmation by both morphology and DNA analysis. Images were obtained using a high-resolution microscope camera (s) (HRMC) (OLYMPUS DP12 and DP20) with 3D software (OLYMPUS Cellsens Standard).

### Molecular confirmation of beetle identity

In addition, DNA from one female specimen (voucher SAX551) was extracted and amplified via PCR and sequenced partial cytochrome oxidase I (COI) (675 bp) and carbamoyl-phosphate synthetase 2, aspartate transcarbamylase, and dihydroorotase (CAD) (501 bp) following the protocol in Cognato et al.^45^. Sequences (COI: OR822285; CAD: OR827366) were subject to a Blast search in GenBank which returned similar *Euwallacea* sequences generated by Wang et al.^31^. These sequences (COI: MN619931, MN619937, MT026213, MT623419-MT62341951, MT623453-MT623457; CAD: MN620205, MN620212, MT634154-MT634186, MT634188-MT634192) were assembled into a Nexus file for phylogenetic analysis. In PAUP*^46^, a heuristic search with 100 stepwise random additions was used to find the most parsimonious trees. Bootstrap values were calculated with 300 pseudoreplicates using heuristic searches with simple addition.

### Fungal isolation from the adults and culturing

Potato dextrose agar (PDA: 4 g potato starch, 20 g dextrose, 15 g agar, distilled water up to 1 L) supplemented with streptomycin sulfate (100 mg/L), and synthetic low-nutrient agar (SNA: 1 g KH_2_PO_4_; 1 g KNO_3_; 0.5 g MgSO_4_·7H_2_O; 0.5 g KCl; 0.2 g Glucose; 20 g Agar; 1 L distilled water) were autoclaved at 121 °C for 15 min. Sterile 9-cm Petri dishes (INA-OPTIKA Co., Ltd., Osaka, Japan) containing 10-ml PDA or SNA culture medium were prepared and kept in a sterile laminar flow chamber under UV light until culture medium solidification. Fungal cultures on PDA were used to characterize colony and odor, whereas those on SNA were used to examine microscopic characters.

Whole beetles were surface-washed by vortexing for 15 s in 1.5-mL sterile tubes containing 1 mL sterile distilled water and one small drop of Tween 20 (< 10 μL). The surface-washed beetles were dried on sterile filter paper after rinsing with sterile distilled water. Each beetle sample was separated into head, thorax, and abdomen under a dissection microscope with two sterilized pins. Afterward, the three body parts without being ground up were singly inoculated on PDA plates at 25 °C for 7–10 days. The total number of fungal colonies formed on each plate was recorded, and the colonies were transferred to other PDA plates for purification.

### Fungal identification and quantification

Using the method described by Jiang et al.^47,48^, the purified isolates from each body part were initially sub-identified at the morphotype level, based on colony properties (e.g., color, thickness, transparency, texture, and growth speed) and fungal micro-structures. They were observed and photographed using a compound microscope (OLYMPUS BX41) equipped with HRMC (OLYMPUS DP23). Five isolates of each morphotype were stored on PDA slants at 25 ℃.

At least one isolate from each morphotype was selected for DNA extraction. Final isolate identification was based on the sequencing of internal transcribed spacer (ITS) rDNA, adding three genes for *Fusarium* fungi: translational elongation factor 1-α (TEF1), DNA-directed RNA polymerase II largest (RPB1), and second largest subunit (RPB2) (Fig. 2). These sequences were amplified using primer pairs, ITS1F and ITS4 for ITS sequences^49^, EF1 and EF2 for TEF1^50^, AF-RPB1F and AF-RPB1R for RPB1^47^, and AF-RPB2F and AF-RB2R for RPB2^47^. Amplicons were purified and sequenced as described by Jiang et al.^47,48^. A homology search was performed with each obtained sequence on the web site of NCBI (https://blast.ncbi.nlm.nih.gov/Blast.cgi) and used together with morphological characters to identify isolated fungi (Supplementary Table S1). For the identification of ambrosia fusaria, phylogenetic analysis was conducted with related *Fusarium* spp. (Fig. 2, Supplementary Table S2). The phylogeny tree was constructed using the maximum likelihood method based on the Kimura 2-parameter model with MEGA7^51^. The tree involved 70 nucleotide sequences. All positions with < 95% site coverage were eliminated; fewer than 5% alignment gaps, missing data, and ambiguous bases were allowed at any position. A total of 1,745 positions were present in the final dataset.

RD and FO of fungal species isolated from each body part were calculated using the equations below:

1 $$\mathrm{RD }\left(\mathrm{\%}\right)=\frac{\mathrm{Number\,of\,fungal\,isolates\,of\,each\,species}}{\mathrm{Total\,number\,of\,fungal\,isolates\,of\,all\,the\,species}}\times 100$$

2 $$\mathrm{FO }\left(\mathrm{\%}\right)=\frac{\mathrm{Number\,of\,beetles\,from\,which\,each\,fungal\,species\,was\,isolated}}{\mathrm{Total\,number\,of\,the\,beetles\,used\,for\,isolation}}\times 100$$

### Pathogenicity tests

A total of 30 mango saplings (1-year-old) were used for this test (Table 3). The variety ‘Irwin’ of *M. indica* was selected because the cultivated trees of this variety commonly show severe dieback symptoms in mango orchards of Japan. All experimental research and field studies involving plants, whether cultivated or wild, including the collection of plant material, adhered to relevant institutional, national, and international guidelines and legislation. The methods employed were in accordance with the appropriate guidelines, regulations, and legislation. The plant material was sourced as follows:

**Table 3:** Inoculation of *Mangifera indica* saplings with different isolates.

Treatment	Test code	No. of saplings	Inoculum	
			Source	Morphology
Wound inoculation	FK	10	*Fusarium kuroshium*^a^	Hyphae
	FD	10	*Fusarium decemcellulare* ^b^	Hyphae
Control	CT	10	Sterilized toothpicks ^c^	–

^a^Isolate from the head of an adult female *E. fornicatus* on October 4, 2018.

^b^Isolate from the abdomen of an adult female *E. fornicatus* on October 5, 2018.

^c^Wounded control without fungal inoculum.

Nature of Biological resource: Plant.

Common Name: Mango.

Scientific Name (Genus and Species): *Mangifera indica*.

Exact part used: Stem.

Source of access (Wild/Culture/Trader): Trader.

Exact place (village, Taluk, District, State) of access of Biological source: Nursery tree growers (members of Japan Agricultural Cooperatives).

All saplings were acquired from a nursery stock of the growers and transferred to the greenhouse of Nagoya University (Nagoya, Japan) on May 21, 2021. After two weeks, the saplings were transferred to plastic bags filled with potting media (Super soil, Akimoto Tensanbutsu Co. Ltd., Mie, Japan), and each pot was covered with a fine nylon net to prevent the entry of root feeders (scarab beetle). None showed disease symptoms until the beginning of fungal inoculation.

To resolve issues associated with high air temperatures during the growth period of saplings, a black sunshade net was installed inside the greenhouse. A data logger (Thermochron G type, KN laboratories Inc., Osaka, Japan) was set 1.2 m above the floor to monitor the air temperature within.

*F. kuroshium* (EF-1-H) and *F. decemcellulare* (EF-44-A), which were isolated from *E. fornicatus*, were designated as fungal inocula in this test (Table 3). They were grown on PDA media in a 9-cm Petri dish for 3 weeks (25 ℃, dark). At the same time, ten sterilized toothpicks (L = 7 cm, d = 2.2 mm) were added to each dish to adhere to their hyphae. Sterilized toothpicks were used as the control inoculum. Before inoculation, four sets of holes (4 mm diameter) were made on the stem (1.15 ± 0.12 cm, diameter; mean ± SD) (Supplementary Table S3) of each sapling by vertically drilling through the center of the stem with an electric drill, starting at 5 cm above soil surface with 2 cm intervals between holes (Supplementary Fig. S2).

Fungal inoculation was conducted on September 9, 2021, as previously described by Jiang et al.^52^. Toothpicks contained with *F. kuroshium* (FK) and *F. decemcellulare* (FD) were inserted into the holes of each of 10 mango saplings (Table 1). CT were also inserted into the holes of 10 saplings. Immediately after inoculation, each inoculation site was sealed with paraffin tape (Parafilm) to prevent dehydration.

### Monitoring after inoculation

All inoculated saplings were examined daily for external symptoms until October 21, 2021. The maximum air temperature in the greenhouse during this test period was 31.42 ± 4.29 °C.

Classification of the wilting process was defined as follows: i) no symptoms (NS): no apparent difference compared to non-inoculated saplings; ii) leaf wilting (LW): some leaves began to droop and wilt; iii) browning of leaves and discoloration of branches (BD): almost all leaves became brown and branch discolored; iv) sprouting of shoot (SS): some shoots sprouted below inoculation part (Supplementary Fig. S3).

LSC was measured in *m*mol/m^2^/s units during 9:00–13:00 h on sunny days (on days when rain was forecast, measurement was moved to an earlier or later time) twice a week, using a leaf porometer (SC1-Leaf Porometer, Decagon Devices Inc., Pullman, WA, USA). Each sapling was watered using 400–500 mL of water between 16:00–17:00 h of the previous day. Five leaves in each sapling were selected randomly and numbered with tags in ascending order, to fix the measurement order of all leaves. The five data points for each sapling were averaged.

### Xylem sap-conduction test

After monitoring external symptoms in the inoculated saplings, some were evaluated for water-flow in their main stems (FK = 10, FD = 10, CT = 6 saplings) (Supplementary Fig. S4). Immediately after being cut at the base of the main stems, the cut ends were immersed in 1% (*w/v*) aqueous acid fuchsin for 5 h in the greenhouse. Next, the main stems were cut into 5-cm long segments. The xylem sap-conduction area (pink area, dyed with acid fuchsin; functional xylem), the xylem discoloration area (brown area, reacted to drilling injury and mycelial infestation; non-functional xylem), and the whole cross-section area (excluding the pith) (Supplementary Fig. S5) of the cut ends of these segments were measured. Images of the cut ends were obtained via a digital camera (Olympus Tg-3), and each area was estimated using ImageJ (Win64, version 1.53f51, National Institutes of Health, USA).

XS or XD in each segment was calculated as follows:

3 $$\mathrm{XS }\left(\mathrm{\%}\right)=\frac{\mathrm{Pink\,area }}{{\text{Cross-section area}}}\times 100$$

4 $$\mathrm{XD }\left(\mathrm{\%}\right)=\frac{\mathrm{Brown\,area}}{{\text{Cross-section area}}}\times 100$$

### Lesion length measurement and re-isolation of inoculated fungi

After obtaining images of cut ends for the xylem sap-conduction test, the segments were used to measure lesion (brown-colored wood tissues) lengths in longitudinal and tangential directions (Fig. 6a,b) and for the re-isolation of inoculated fungi, including additional segments of CT-01 sapling (Supplementary Fig. S4).

An upper 1 cm section was cut off from each 5-cm long segment and longitudinally divided into two parts using a tree pruner. The two parts were immediately surface-sterilized for 1 min in 70% (*v/v*) ethyl alcohol solution. A second sterilization was performed using 1% (*v/v*) antiformin solution for 1 min. A rinse wash was performed using sterile distilled water for 1 min followed by drying using sterile filter paper for 1 min.

The sterilized section parts were placed on PDA (25 ℃, dark), and re-isolated fungi were confirmed via colony morphology and DNA sequencing methods described above.

The remaining 4-cm long segment was also divided longitudinally into two parts using a tree pruner. The cross-sections were photographed for the purpose of detecting lesions spreading around the inoculation site (Fig. 6a,b). ImageJ was used to measure the lesion lengths in the photographs.

### Statistical analyses

Differences in the median FOs among adult body parts or the fungal species were compared using Fisher’s exact test. Lesion lengths in inoculation treatments were compared using the Kruskal–Wallis test. Multiple comparisons among them were conducted using the post-hoc tests with Bonferroni correction. Analyses were performed using SPSS version 19.0 software (IBM Corporation, Armonk, NY, USA, 2010).

### Supplementary Information

Supplementary Information 1.

Supplementary Information 2.

Supplementary Information 3.

Supplementary Information 4.

## Data Availability

All data generated or analyzed during this study are included in this published article and its Supplementary Information files.
