# Supplementary material for: Fusarium kuroshium is the primary fungal symbiont of an ambrosia beetle, Euwallacea fornicatus, and can kill mango tree in Japan
Source: Sci Rep. 2023 Dec 7;13:21634. doi: 10.1038/s41598-023-48809-8 (PMC10703777; doi:10.1038/s41598-023-48809-8)
Supplement: Supplementary file 1 — Supplementary Information 1. [file 41598_2023_48809_MOESM1_ESM.docx]

*Fusarium kuroshium* is the primary fungal symbiont of an ambrosia beetle, *Euwallacea fornicatus*, and can kill mango tree in Japan

Zi-Ru Jiang^1*^, Momo Tanoue^2^, Hayato Masuya^3^, Sarah M. Smith^4^, Anthony I. Cognato^4^, Norikazu Kameyama^5^, Keiko Kuroda^6^, Hisashi Kajimura^1^**^*^**

^1^Laboratory of Forest Protection, Graduate School of Bioagricultural Sciences, Nagoya University, Nagoya 4648601, Japan.

^2^School of Agricultural Sciences, Nagoya University, Nagoya 4648601, Japan.

^3^Department of Forest Microbiology, Forestry and Forest Products Research Institute, Tsukuba 3191301, Japan.

^4^Department of Entomology, Michigan State University, MI 48824, USA.

^5^Faculty of Agriculture, University of the Ryukyus, Okinawa 9030213, Japan.

^6^Graduate School of Agricultural Science, Kobe University, Kobe 6570013, Japan.


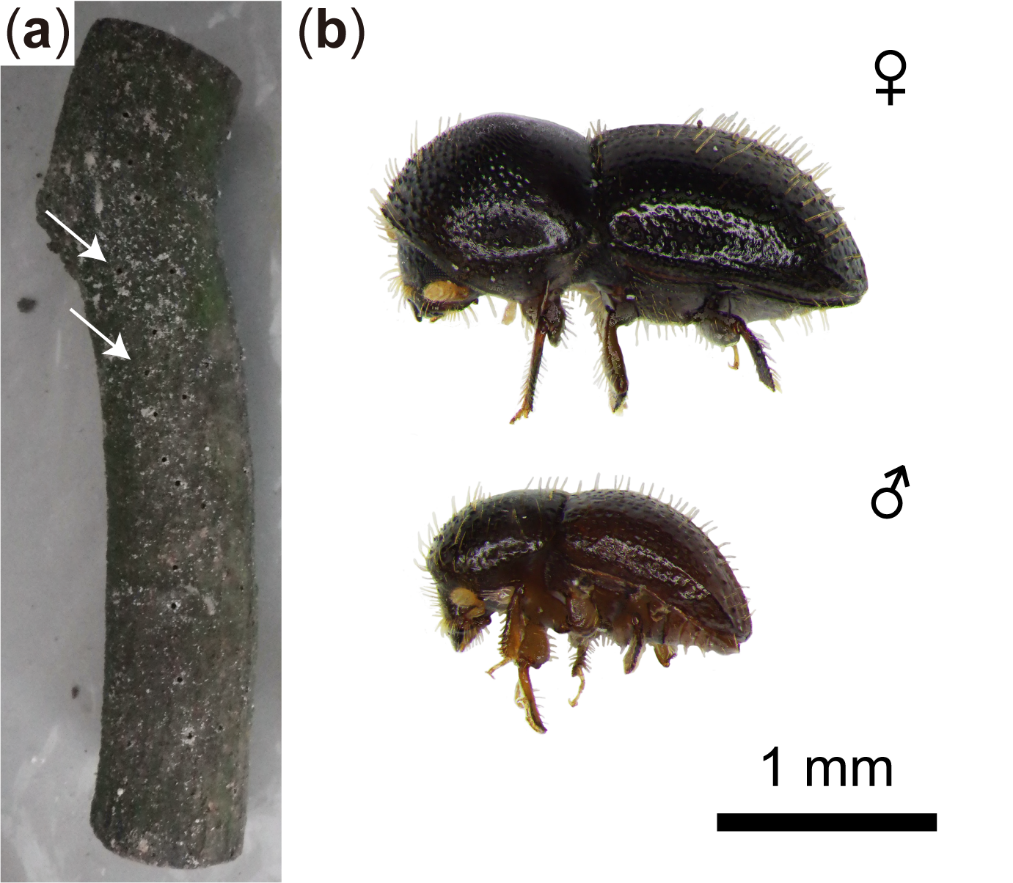


**Figure S1. Female adults of ambrosia beetle (*Euwallacea fornicatus*)-infesting mango tree (*Mangifera indica*) branches.** (a), A mango tree branch collected at Nago city, Okinawa main Island, on August 30, 2018, with many bored holes (white arrow: two holes of them); (b), Lateral views of adult *E. fornicatus* emerged from the branch (a).


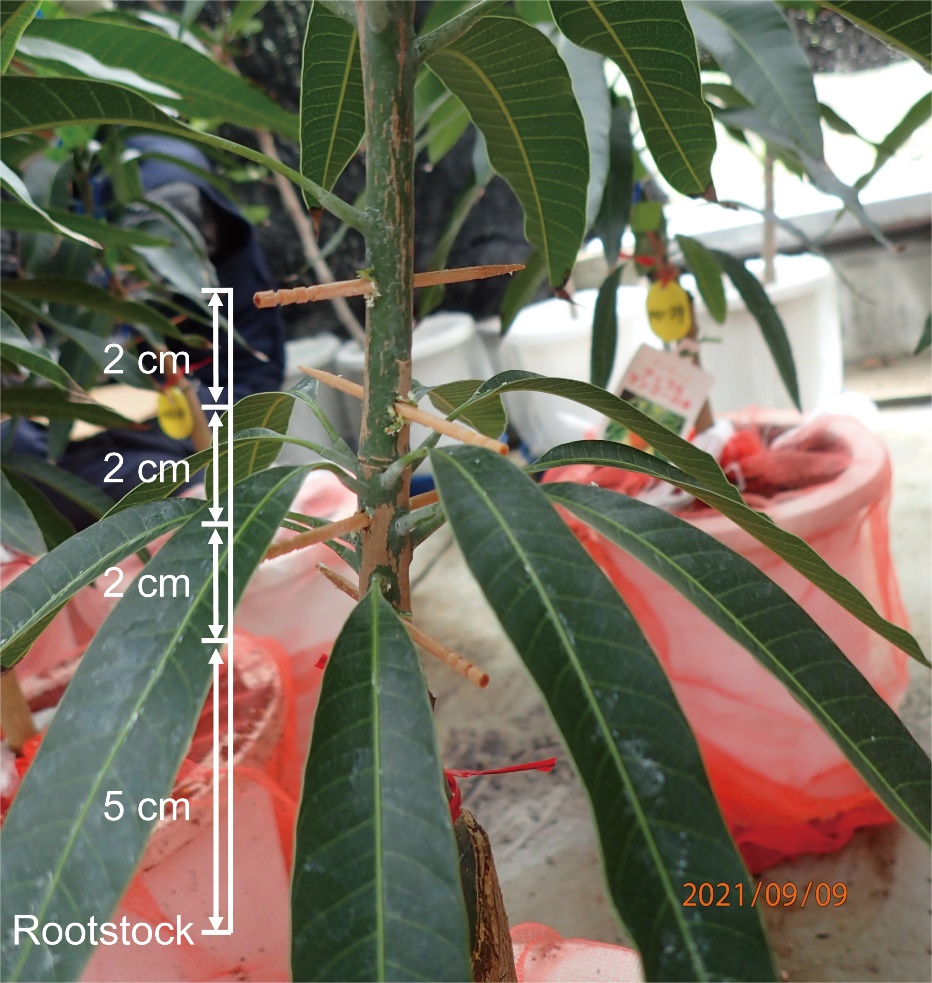


**Figure S2. Inoculation of healthy *Mangifera indica* sapling with toothpicks containing mycelia of the isolated *Fusarium* species.** Four toothpicks were used to pierce the stem at 2 cm intervals between them, starting at 5 cm above soil surface of the sapling.


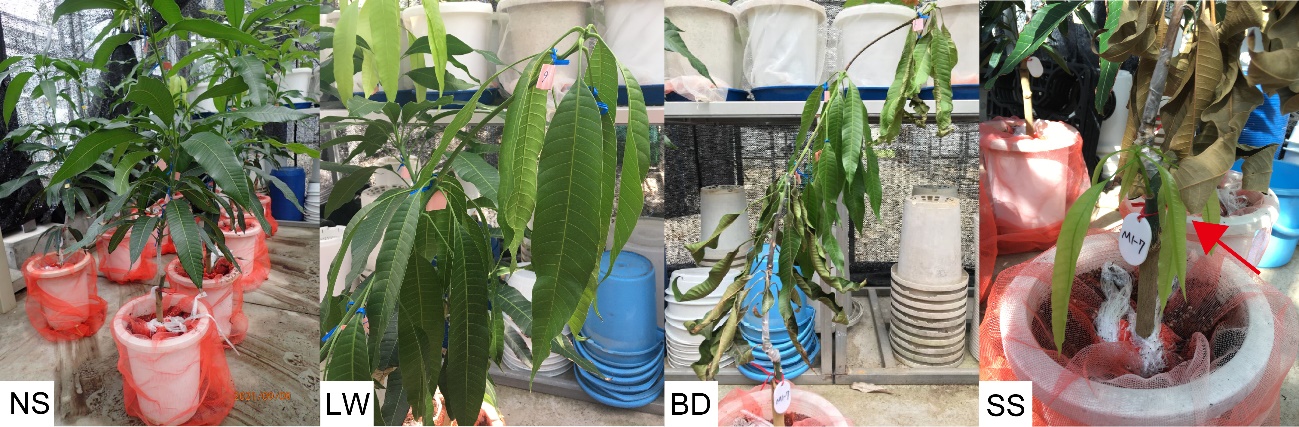


**Figure S3. External symptoms observed in *Mangifera indica* sapling inoculated with *Fusarium kuroshium*.** Images of the No.7 sapling (FK_07) are shown. NS, No symptoms (healthy leaves and stem) on day of inoculation; LW, Leaf wilting 3 d after inoculation; BD, Browning of leaves and discoloration of branches 12 d after inoculation; SS, sprouting of shoot (red arrow) below inoculation part 35 d after inoculation.


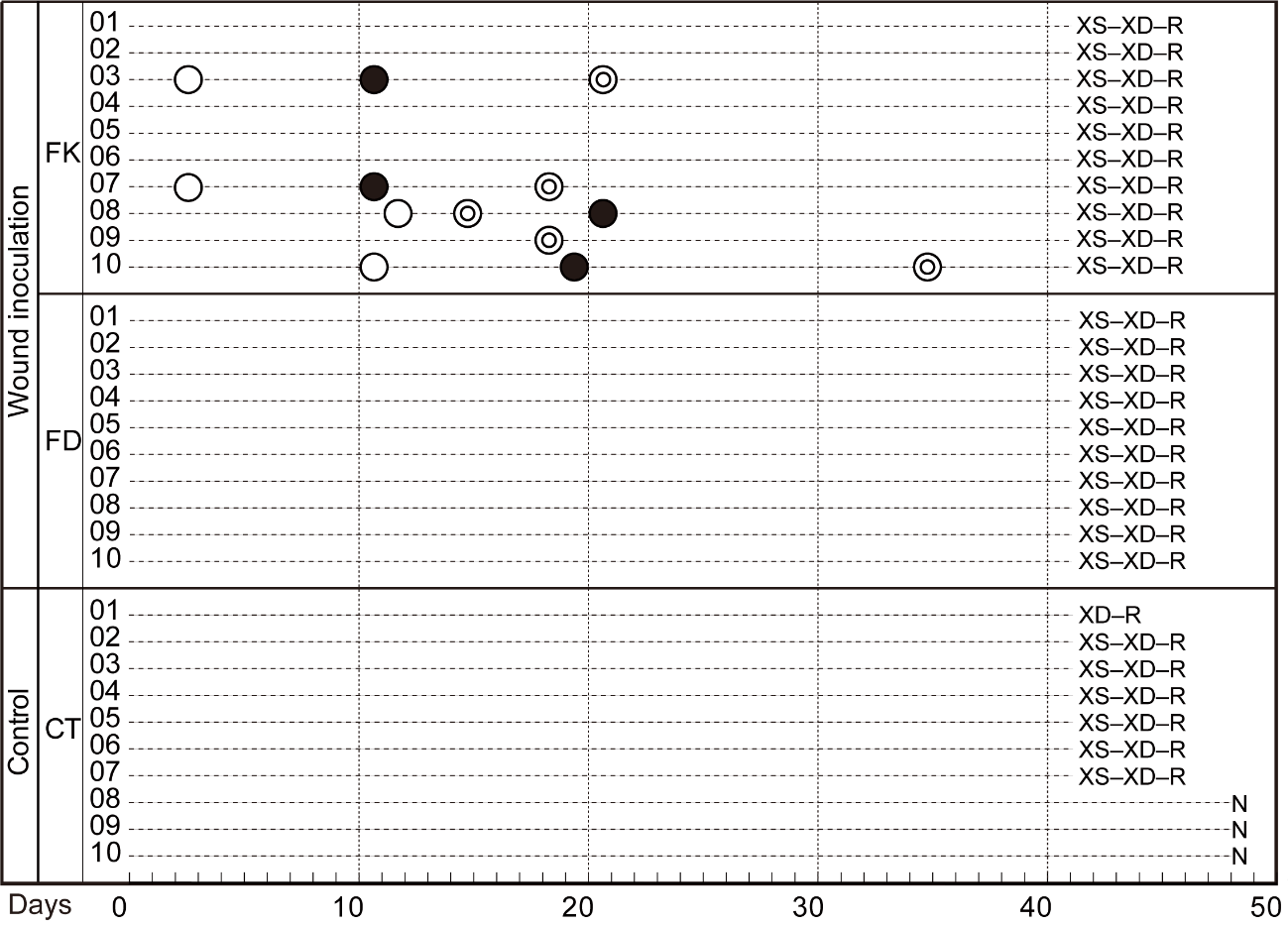


**Figure S4. Development of external symptoms in each *Mangifera indica* sapling during the observation period following inoculation.** Sterilized toothpicks were used to inoculate the saplings numbered 01–10 in sections labeled FK, FD and CT with *Fusarium kuroshium* and *Fusarium decemcellulare*, isolated from *Euwallacea fornicatus* (Table 2), respectively. Open circle: start of leaf dropping; Filled circle: browning of all leaves and dead of stem; Double circle: sprouting below inoculation part; XS: xylem sap-conduction test; XD: xylem discoloration measurement; R: re-isolation of inoculated fungi; N: no tests were conducted.


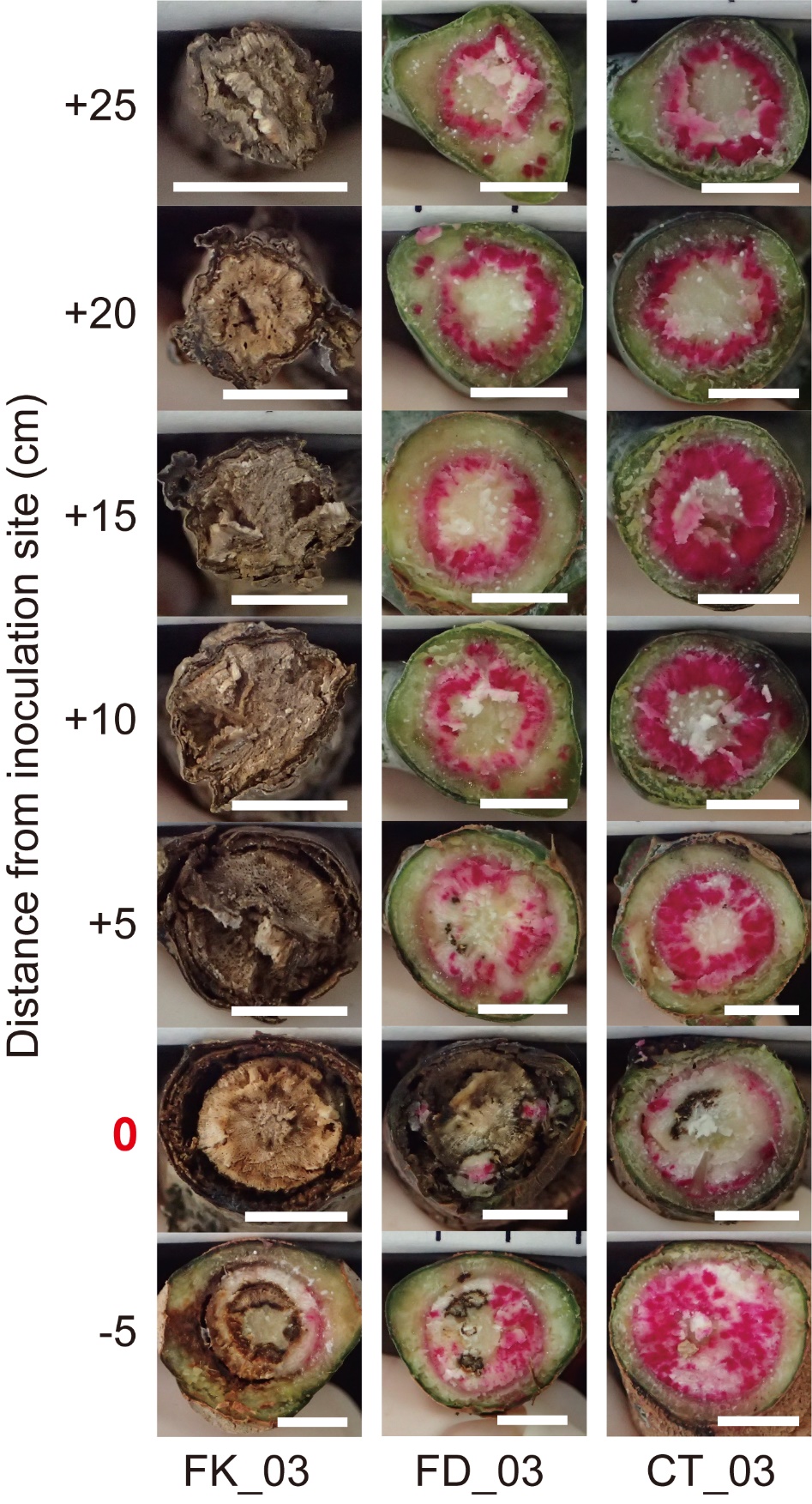


**Figure S5. Surfaces of stem crosscuts of *Mangifera indica* saplings, showing absorbed acid fuchsin solution.** Images are shown with white scale bars 5 mm long. See the footnote in Figure S4 for code of the saplings.
